# Ocular biometric parameters of mild hyperopia to mild myopia children aged 6–14 years from Wenzhou optometry center: A cross-sectional study

**DOI:** 10.3389/fmed.2022.992587

**Published:** 2022-10-06

**Authors:** Weiqin Liu, Weishai Liu, Chenxiao Wang

**Affiliations:** ^1^School of Ophthalmology and Optometry and Eye Hospital, Wenzhou Medical University, Wenzhou, China; ^2^Department of Ophthalmology, Ankang Hospital of Traditional Chinese Medicine, Ankang, China

**Keywords:** lens power, myopia, refractive error, ocular biometric parameters, corneal curvature

## Abstract

**Introduction:**

Myopia is the most common visual disorder in school-aged children and adolescents worldwide. This study aimed to explore the ocular biometric characteristics of children aged 6–14 years from the Wenzhou optometry center and to determine the relationship between spherical equivalent refraction (SER) and macular pigment optical density (MPOD).

**Subjects and methods:**

Participants underwent a full-scale ophthalmic examination anteriorly and posteriorly. Relevant parameters were documented, such as axial length (AL), anterior chamber depth (ACD), SER and lens thickness (LT), corneal curvature radius (CCR), and MPOD. Lens power (LP) was calculated using Bennett’s formula. Shapiro–Wilk tests and histograms were used to check the normality of the distribution of refractive and ocular biometric parameters. Scatter diagrams were adopted to analyze the relationships between refraction and parameters of ocular biometry. Multiple linear regression models were employed to fit the associated factors of AL, AL/CCR, and LP.

**Results:**

A total of 902 mild hyperopia to mild myopia (+3.00 D ≤ SE ≤ −3.00 D) children aged 6–14 years were included. The mean age of participants was 10.03 ± 2.47 years, and the prevalence of mild hyperopia, emmetropia, and myopia was 5.65, 27.05, and 67.30%, respectively. The prevalence of mild myopia increased from 30.53% at 6 years of age to 93.62% at 14 years of age. Overall, AL, ACD, and AL/CCR increased, but LP declined from 6 to 14 years of age, whereas CCR and MPOD remained stable. An increase of 1 mm in AL was associated with −0.69 D of myopic change. A unit increase in AL/CCR was associated with −7.87 D in SER. As for the SER variance, AL explained 30.5% and AL/CCR explained 51.1%, whereas AL/CCR and LP accounted for 59.2%.

**Discussion:**

In this work, we have studied the distributions of ocular biometric characteristics of mild hyperopia to mild myopia children from the perspective of an optometry center rather than a sampling survey. In addition, we found that children from the optometry center had a slower progression toward myopia than those from previous sampling surveys, which was an informative finding for future myopia prevention. In addition, we have made a correlation analysis between the macular pigment optical density and spherical equivalent refraction. Though, no correlation was found.

## Introduction

The “epidemic” of myopia has overtly soared in the past few decades (1). Over 2.6 billion people around the world are presently beset by eye ailment, and the population size is expected to reach three billion by the end of this decade and four billion by 2050 (2, 3). The prevalence of myopia in China, accounting for some 40% of the population (about 600 million people), is still far higher than that in India and Nepal as well as other countries beyond East Asia (4–6). The proportions of myopia and high myopia, meanwhile, are mounting up year by year (2). A newly-published article (7) indicated that the prevalence of myopia in primary, secondary, and high school is, respectively, 38.16, 77.52, and 84.00%, and that high myopia incidence makes up 0.95, 6.90, and 12.98% at each educational stage in China. As a rule of thumb, an individual with high myopia is more likely to develop pathologic myopia than someone with mild myopia (8). A series of disturbing complications could be followed in patients with pathologic myopia, such as glaucoma, cataract, retinal detachment, myopic choroidal neovascularization, and blindness (9). In terms of social and economic costs of myopia, the direct economic cost of correcting myopia, as reported, is at least US$3.8 billion annually and the potential myopia-related global loss of productivity is US$244,000 million (10).

The causes of myopia have been extensively studied. Environmental and genetic factors as well as habits of using eyes are believed to contribute to the development of myopia (1). It is still hard to answer why the axial length (AL) of patients with myopia keeps elongating, even moving into the third decade of their life (11), although a few strategies for managing and controlling myopia have made some progress (3).

Ametropia indicates a mismatch between the focal length and the axial length of the eye. The precise mechanism of how ocular components, such as the cornea, AL, and lens coordinate in the process of emmetropization is not fully understood. The emmetropic visual feedback model suggests that the defocusing effect can retard refractive errors, which can be described as an “active” mechanism (12). The establishment of refractive development archives (RDA) is considered to be a promising measure to understand the occurrence and developmental trajectory of myopia, and then to find effective prevention and control measures to curb the trend of myopia (13).

The macular pigment has been the focus of much attention in recent years, due to its protective effect against macular degenerations (14). There is a growing interest in the pathophysiological implications of macular pigment optical density (MPOD) with respect to ocular pathologies, such as age-related macular degeneration (AMD) (15, 16), chorioretinopathy, and retinitis pigmentosa. However, to the best of our knowledge, it is yet to be determined whether MPOD is associated with the pathogenesis of myopia in youth. Little attention has been paid to the relationship between MPOD and spherical equivalent refraction (SER), even though SER is one of the most important parameters of the visual system. Therefore, the purpose of our study is to explore the ocular biometric characteristics of children aged 6–14 years from the Wenzhou optometry center and to determine the relationship between spherical equivalent refraction (SER) and macular pigment optical density (MPOD).

## Subjects and methods

A total of 1,072 children or adolescents aged 6–14 years from the optometry center of Eye Hospital of Wenzhou Medical University participated in this cross-sectional study, which was performed with abidance by the Declaration of Helsinki and approved by the Ethics Committee of Eye Hospital of Wenzhou Medical University. Written and verbal informed consent was acquired from all parents or guardians. All participants underwent a full-scale ocular examination administered.

Measurement parameters, such as AL, anterior chamber depth (ACD), and corneal curvature radius (CCR), were obtained from the optical low coherence reflectometry (OLCR) based Lenstar with cycloplegia (Ls900, Haag-Streit, Swiss). The crystalline lens power (LP) is of great concern in the growth and development of the eyeball in its natural state. LP and AL-to-CCR ratio (AL/CCR) were calculated, of which LP was calculated using the Bennett formula. The digital fundus camera system (VISUCAM 224; Carl Zeiss Meditec, Oberkochen, Germany) was used to record fundus photography and measure the macular pigment optical density (MPOD). Other examinations included intraocular pressure, best-corrected visual acuity, and assessment of anterior and posterior segments with the aid of slit-lamp and the HD-optical coherence tomography (HD-OCT5000, Carl Zeiss Meditec Inc., Dublin, CA, USA), respectively.

The inclusion criteria for participants were as follows: (1) children aged 6–14 years; (2) IOP less than 21 mmHg; (3) normal anterior chamber; (4) the absolute value of SER less than or equal to 3.0 D; (5) best corrected visual acuity (BCVA) not less than 1.0; and (6) normal fundus appearance. Participants with a history of disease were excluded, such as hypertension, diabetes, congenital cataract, congenital glaucoma, intraocular injections or surgery, refractive surgery, and other clues of oculopathy were also eliminated by trained ophthalmologists and questionnaires. Spherical equivalent refraction was calculated with the formula: SER = half of the cylindrical power plus the spherical power. Cylindrical power and spherical power were obtained under subjective refraction, which was performed by an experienced optometrist. Mild hyperopia was defined as + 0.50 D ≤ SE ≤ + 3.00 D; emmetropia was defined as –0.50 D < SE < + 0.50; and mild myopia was defined as –3.00 D ≤ SE ≤ –0.50 D.

Biometric and refractive parameters were analyzed and plotted as a function of age and gender. Data were analyzed on the computer using the software SPSS 22.0 (IBM SPSS Inc., Chicago, IL, USA). Shapiro–Wilk tests and histograms were used to check the normality of the distribution of refractive and ocular biometric parameters. An independent sample *t*-test and trend analysis were performed to detect the gender and age differences, respectively. The statistical significance was uniformly defined as *p* < 0.05 (two-sided). The Spearman correlation coefficient was used to indicate a correlation between the left and right eyes. A multiple linear regression analysis was conducted to explore the associations between refraction and parameters of ocular biometry.

## Results

A total of 1,072 school-aged students registered for this population-based study. Of the enrolled participants, 168 failed to meet the inclusion criteria, two dropped out due to unsuccessful biometric measurements. Therefore, 902 students aged 6–14 years were selected for further statistical analysis. The research population included 476 male and 426 female children, with a mean age of 10.03 ± 2.47 years. Both the right and left eyes’ data were analyzed in the current study. Due to the high correlation between the right and left eyes of the same individual, only data from the right eye were presented.

**FIGURE 1:**
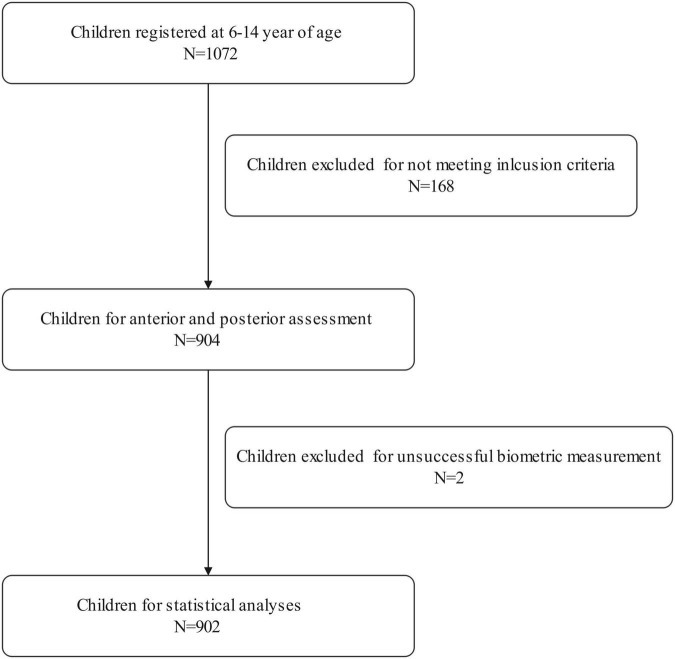
A flowchart depicting the inclusion of participants assessed in this study.

**FIGURE 2:**
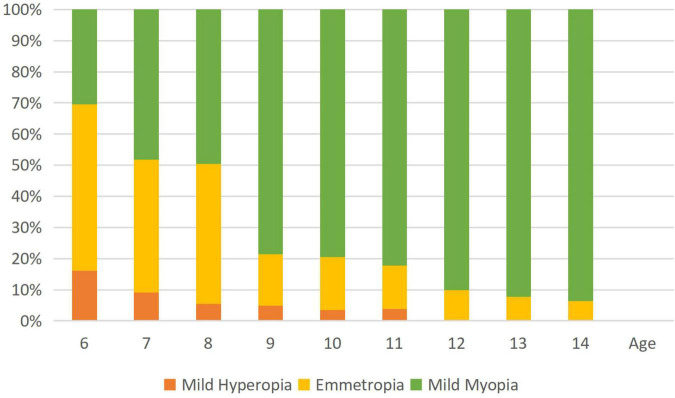
The histogram shows age-specific distributions of the prevalence of refractive errors (mean spherical equivalent in diopters) in the study population. Mild hyperopia is defined as +0.50 D ≤ spherical equivalent refraction (SER) < +3.00 D; emmetropia is defined as –0.50 D < SER < +0.50; and mild myopia is defined as –3.00 D ≤ SER < –0.50 D.

**FIGURE 3:**
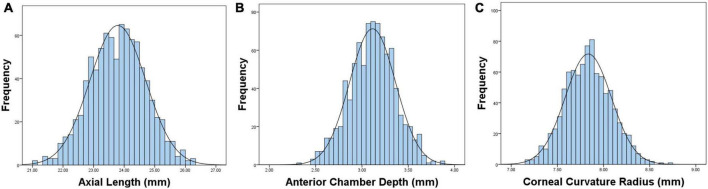
Graphs showing the distributions and fitted normative curves of ocular biometric parameters: axial length (AL) **(A)**, anterior chamber depth (ACD) **(B)**, corneal curvature radius (CCR) **(C)**.

**FIGURE 4:**
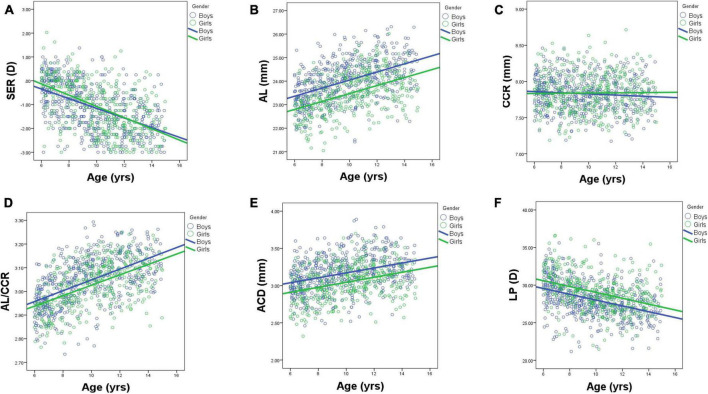
Scatterplots showing individual data for ocular parameters: SER **(A)**, AL **(B)**, CCR **(C)**, LPAL/CCR **(D)**, ACD **(E)**, and LP **(F)** by age and gender. AL, axial length; ACD, anterior chamber depth; CCR, corneal curvature radius; LP, lens power.

**FIGURE 5:**
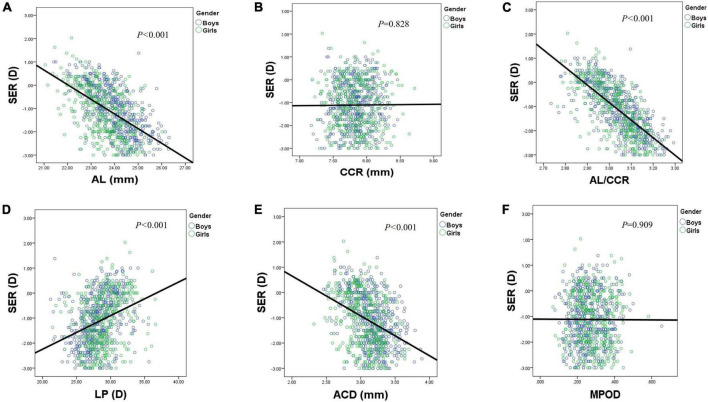
Scatterplots showing individual data for SER by AL **(A)**, CCR **(B)**, AL/CCR **(C)**, LP **(D)**, ACD **(E)**, and MPOD **(F)**. AL, axial length; ACD, anterior chamber depth; CCR, corneal curvature radius; LP, lens power; MPOD, macular pigment optical density.

Table 1 shows the prevalence of different refraction errors in each group, and Figure 1 shows the flowchart depicting the inclusion of participants assessed in this study. Figure 2 shows the distribution of different refractive status in different age groups. The prevalence of mild hyperopia and emmetropia showed a decreasing trend from 16.03 and 53.44% at the age of 6 years to 0 and 6.38% at the age of 14 years, respectively, while the prevalence of mild myopia increased rapidly from 30.53% at the age of 6 years to 93.62% at the age of 14 years. The average SER of both boys and girls aged 6–14 years showed a descending trend (Figure 4A, *p* < 0.001), from –0.23 ± 0.77 D to –1.99 ± 0.94 D. Statistically significant differences in SER were found between boys and girls in the two age groups (age 8 and 9 years), with boys being more myopic SER than girls. However, most groups made no statistical difference. The detailed distribution of SER by age and gender is shown in Table 2. The ALs of right eyes were normally distributed as shown in Figure 3A. The correlation between the ALs of left and right eyes was very high, with a coefficient of 0.92. There were significant differences in AL between boys and girls in all the nine groups. At all ages, the AL of boys was significantly longer than that of girls (all *p* < 0.05). Using the data as a reflection of longitudinal changes, AL was elongated at a rate of 0.19 and 0.18 mm per year for boys and girls, respectively, from 6 to 14 years old (Figure 4B). On an average, a 1 mm increase of AL was associated with a –0.69 D change of SER (Figure 5A). The distribution of anterior chamber depth is normal as shown in Figure 3B.

**TABLE 1:** The prevalence of mild hyperopia, emmetropia, and mild myopia children of 6–14 years of age.

		Mild hyperopia			Emmetropia			Mild myopia		
Age	All.	No.	%	95%CI	No.	%	95%CI	No.	%	95%CI
All	902	51	5.65	3.89–6.6.92	244	27.05	24.62–30.55	607	67.30	65.81–71.95
6	131	21	16.03	10.42–23,69	70	53.44	50.60–67.92	40	30.53	27.82–44.78
7	110	10	9.09	4.69–16.48	47	42.73	33.46–52.52	53	48.18	38.63–57.86
8	129	7	5.43	2.40–11.28	58	44.96	36.28–53.95	64	49.61	40.74–58.50
9	103	5	4.85	1.80–11.50	17	16.50	10.18–25.39	81	78.64	69.24–85.86
10	117	4	3.42	1.10–9.04	20	17.09	10.99–25.41	93	79.49	70.83–86.17
11	107	4	3.74	1.21–9.85	15	14.02	8.32–22.39	88	82.24	73.40–88.70
12	81	0	0	NA	8	9.88	4.67–19.05	73	90.12	80.95–95.33
13	77	0	0	NA	6	7.79	3.21–16.79	71	92.21	83.21–96.79
14	47	0	0	NA	3	6.38	1.66–18.56	44	93.62	81.44–98.34

No., number; N/A, not applicable. Mild hyperopia is defined as +0.50 D ≤ SER ≤ +3.00 D; emmetropia is defined as –0.50 D < SER < +0.50, and mild myopia is defined as –0.50 D ≤ SER ≤ –3.00 D.

**TABLE 2:** Refractive and biometric characteristics of the population by gender and age.

	Age (years)									*P* (trend)
	6	7	8	9	10	11	12	13	14	
**SER (D)**										
Total	–0.23 ± 0.77	–0.61 ± 0.94	–0.58 ± 0.80	–1.45 ± 1.03	–1.59 ± 1.16	–1.74 ± 1.17	–1.83 ± 0.99	–1.91 ± 0.95	–1.99 ± 0.94	<0.001
Boys	–0.27 ± 0.72	–0.70 ± 0.95	–0.70 ± 0.81	–1.62 ± 1.06	–1.51 ± 1.20	–1.75 ± 1.09	–1.79 ± 1.07	–1.86 ± 0.91	–1.91 ± 0.91	<0.001
Girls	–0.16 ± 0.87	–0.48 ± 0.93	–0.39 ± 0.75	–1.22 ± 0.96	–1.68 ± 1.11	–1.74 ± 1.25	–1.89 ± 0.89	–1.99 ± 1.02	–2.09 ± 0.99	<0.001
*P*-value	0.497	0.251	0.030	0.048	0.414	0.966	0.634	0.562	0.484	
**AL (mm)**										
Total	23.06 ± 0.74	23.39 ± 0.82	23.51 ± 0.83	23.96 ± 0.83	24.08 ± 0.87	24.16 ± 0.82	24.40 ± 0.86	24.48 ± 0.84	24.52 ± 0.93	<0.001
Boys	23.25 ± 0.67	23.70 ± 0.71	23.83 ± 0.75	24.28 ± 0.70	24.23 ± 0.95	24.47 ± 0.75	24.60 ± 0.86	24.70 ± 0.81	24.75 ± 0.84	<0.001
Girls	22.67 ± 0.75	22.94 ± 0.77	23.04 ± 0.73	23.53 ± 0.80	23.91 ± 0.73	23.91 ± 0.81	24.14 ± 0.81	24.23 ± 0.68	24.34 ± 0.73	<0.001
*P-*value	<0.001	<0.001	<0.001	<0.001	0.033	<0.001	0.010	<0.001	<0.001	
**ACD (mm)**										
Total	2.96 ± 0.33	3.01 ± 0.25	3.07 ± 0.23	3.14 ± 0.24	3.16 ± 0.26	3.20 ± 0.23	3.25 ± 0.25	3.30 ± 0.24	3.37 ± 0.21	<0.001
Boys	3.00 ± 0.37	3.07 ± 0.24	3.12 ± 0.23	3.19 ± 0.21	3.20 ± 0.30	3.29 ± 0.21	3.33 ± 0.25	3.36 ± 0.23	3.44 ± 0.15	<0.001
Girls	2.88 ± 0.25	2.92 ± 0.23	2.99 ± 0.20	3.07 ± 0.26	3.11 ± 0.20	3.13 ± 0.21	3.16 ± 0.22	3.21 ± 0.22	3.26 ± 0.24	<0.001
*P*-value	0.001	0.001	0.001	0.007	0.056	<0.001	0.001	0.008	0.006	
**CCR (mm)**										
Total	7.85 ± 0.24	7.86 ± 0.27	7.82 ± 0.27	7.83 ± 0.26	7.83 ± 0.24	7.83 ± 0.23	7.83 ± 0.26	7.83 ± 0.25	7.83 ± 0.26	0.356
Boys	7.90 ± 0.24	7.94 ± 0.22	7.90 ± 0.28	7.88 ± 0.28	7.88 ± 0.23	7.89 ± 0.20	7.88 ± 0.27	7.87 ± 0.21	7.93 ± 0.22	0.334
Girls	7.77 ± 0.22	7.74 ± 0.28	7.70 ± 0.23	7.75 ± 0.22	7.77 ± 0.23	7.78 ± 0.25	7.80 ± 0.24	7.75 ± 0.30	7.69 ± 0.24	0.371
*P*-value	0.003	<0.001	<0.001	0.013	0.005	0.012	0.011	0.028	<0.001	
**AL/CCR**										
Total	2.93 ± 0.72	2.98 ± 0.09	3.01 ± 0.08	3.06 ± 0.09	3.08 ± 0.09	3.09 ± 0.10	3.12 ± 0.09	3.14 ± 0.08	3.16 ± 0.09	<0.001
Boys	2.95 ± 0.71	2.99 ± 0.09	3.02 ± 0.09	3.08 ± 0.09	3.07 ± 0.11	3.10 ± 0.10	3.13 ± 0.08	3.15 ± 0.07	3.17 ± 0.07	<0.001
Girls	2.92 ± 0.71	2.96 ± 0.09	2.99 ± 0.08	3.04 ± 0.09	3.08 ± 0.08	3.07 ± 0.09	3.10 ± 0.10	3.13 ± 0.08	3.14 ± 0.10	<0.001
*P*-value	0.027	0.221	0.103	0.007	0.763	0.075	0.095	0.193	0.137	
**LP (D)**										
Total	30.31 ± 2.69	29.36 ± 2.39	28.56 ± 2.53	28.24 ± 2.25	27.75 ± 2.46	27.75 ± 2.55	26.96 ± 2.43	27.37 ± 2.20	27.44 ± 2.78	0.054
Boys	29.84 ± 2.47	28.96 ± 2.22	27.86 ± 2.52	27.63 ± 1.65	27.74 ± 2.32	26.99 ± 2.87	26.45 ± 2.49	26.69 ± 2.19	26.70 ± 2.20	0.048
Girls	31.27 ± 2.90	29.96 ± 2.53	29.60 ± 2.19	29.06 ± 2.67	27.77 ± 2.62	28.35 ± 2.04	27.62 ± 2.20	28.33 ± 1.85	28.45 ± 3.21	0.061
*P*-value	0.003	0.031	<0.001	0.002	0.935	0.002	0.021	<0.001	0.017	
**MPOD**										
Total	0.27 ± 0.08	0.27 ± 0.09	0.26 ± 0.08	0.27 ± 0.07	0.28 ± 0.08	0.29 ± 0.09	0.27 ± 0.08	0.29 ± 0.07	0.029 ± 0.08	0.680
Boys	0.26 ± 0.09	0.26 ± 0.07	0.25 ± 0.09	0.26 ± 0.07	0.27 ± 0.08	0.28 ± 0.08	0.26 ± 0.08	0.28 ± 0.08	0.28 ± 0.08	0.645
Girls	0.28 ± 0.08	0.29 ± 0.10	0.27 ± 0.08	0.27 ± 0.07	0.29 ± 0.09	0.30 ± 0.09	0.28 ± 0.08	0.30 ± 0.06	0.29 ± 0.07	0.712
*P*-value	0.373	0.076	0.168	0.299	0.126	0.192	0.233	0.255	0.627	

SER, spherical equivalent refraction; AL, axial length; ACD, anterior chamber depth; CCR, corneal curvature radius; LP, lens power; D, diopter; MPOD, macular pigment optical density; *P* < 0.05 is considered statistically significant.

An ACD with an ascending trend was observed (Table 2). There were significant differences in ACD in each group between boys and girls, and the average ACD of boys is 0.11 mm deeper than that of girls. On an average, ACD was deepened at a rate of 0.05 and 0.04 mm per year for boys and girls, respectively (Figure 4E). A 1 mm increase of ACD was associated with a –1.70 D change of SE on average (Figure 5E).

Figure 3C shows the distribution of CCR of all individuals, and the curve is normally distributed. Unlike ACD and AL, CCR remained stable with increasing age (Figures 4C, 5B). However, statistical gender differences were observed in all ages. Overall, boys have a greater CCR than girls. On the whole, the AL/CCR increased with age, in both girls and boys (Figure 4D). The statistical difference in the AL/CR ratio between boys and girls was observed in the population, but it was marginal only at the ages of 6 and 9 years of age. There was a linear correlation between the changes in SER and the AL/CCR ratio. On an average, a unit difference in the AL/CR ratio corresponds to a change in SE of –7.87 D (Figure 5C).

Lens power calculated using the Bennett formula was seen as a downward trend, with –0.41 D being changed a year in boys and –0.40 D a year in girls, respectively (Figure 4F). Girls had a greater LP than boys (*p* < 0.05), and a unit change of LP, averagely, was associated with a 0.13 D change of SE (Figure 5D). No statistically significant difference was observed in MPOD between boys and girls in each group (all *p* > 0.05). No correlation was observed between MPOD and SER (Figure 5F).

Three multiple linear regression models were conducted to investigate the relationships between SER and ocular parameters. When only AL was enrolled in model 1, 30.5% of the variance in SER was explained. About 51.1% of the variance in SER was explained, when AL/CCR was included in model 2. The AL/CCR ratio, along with LP was included in model 3, which explained 59.2% variance in SER (Table 3).

**TABLE 3:** Linear regression models for spherical equivalent refraction (SER) with axial length (AL), AL/corneal curvature radius (CCR), and lens power (LP).

	Model 1			Model 2			Model 3		
Variables	B	*P*-value	R^2^	β	*P*-value	R^2^	β	*P*-value	R^2^
AL (mm)	–0.622	<0001	0.305						
AL/CCR				–7.243	<0001	0.511	–10.226	<0001	0.592
LP (D)							–0.170	<0001	

SER, spherical equivalent refraction; AL, axial length; CCR, corneal curvature radius; LP, lens power; D, diopter.

## Discussion

Ametropia represents a mismatch between the focal length and the AL of the eye. The active mechanism of emmetropization is by regulating axial growth, while the passive mechanism of emmetropization is primarily through the regulation of corneal and lens diopters. Previous studies (17, 18) have found that the distribution of refractive errors under the cycloplegia (atropine) circumstance in adult population obviously deviated from the normal distribution, and it concentrated in emmetropia, but markedly showing a positive skewness toward myopia. Based on these results, ophthalmologists put forward a concept that the components of the eyes are actively adjusted to form a pattern of minimizing refractive errors (19), which was strongly supported by recent studies (20, 21).

In our study, a normal distribution with a mean SER of –1.26 ± 1.02 D was obtained in this cohort of Wenzhou children aged 6–14 years. The mean SER (a descending trend with age) in students aged 6 years and above were all < 0 D, which is similar to that reported by Xu et al. (7). What interests us is that the SER decreased slower than that of general investigation previously reported with a mean of –2.49 D of SER decreased from 6 to 14 years old, while ours was –1.76 D in the same period. Preventive actions or treatments were applied to children from the optometry center if the optometrist thought it was necessary. These interventions included the selection of an optimal pair of glasses (conventional lens, or orthokeratology lens, or defocused incorporated multi-segment lens) and employment of low dose atropine. This may explain the fact that patients with myopia who were diagnosed and treated according to clinical standards have a slower progression of myopia than those who hastily grabbed a pair of glasses from a spectacles store. It must be pointed out that the subjects of this study characterized by better economic conditions and educational level, were recruited from clinic rather than the statistical sampling of the population. This explains the high prevalence rate, but it does not affect the study of ocular biological parameters. We admit that the current research is cross-sectional, potential cohort effects cannot be ruled out, so we need to be cautious when interpreting the results.

The onset and development of myopia among children and adolescents is mainly due to the imbalance between AL elongation and lens power reduction during ocular growth (19). In our study, the AL gradually increases with age, from 23.06 at the age of 6 years to 24.52 at the age of 14 years. These results are consistent with the previously reported results targeting both school-aged and pre-school students, and boys at the same age had longer AL than girls (22–25).

Crystal lens refraction, corneal power, and AL are the main factors affecting refractive status. Studies have reported a stabilization of corneal power within 1–2 years after birth (26, 27). In this study, the corneal radius of curvature and anterior chamber depth are basically Gaussian distributions. We found that the corneal radius of curvature increased from 6 to 7 years old, but remained stable after 7 years old, and it came to a near plateau. We speculate that 7 years old could be a cut-off point of corneal stability. Many compensated adjustments of optical components seem to involve refraction. Corneal curvature is a key clinical endophenotype that reflects the refractive status of the eye. Changes in the corneal curvature can significantly lead to refractive errors.

The AL/CCR ratio is undoubtedly an important anatomical signal in the development of myopia in children and adolescents (28). A lot of evidence showed that the AL/CCR ratio had an advantage in correlation with refractive errors over axial length alone (21, 28–30). The AL/CCR ratio of Australians (21) was 2.906, that of Singaporeans (30) exceeded 3.0, and that of the COMET study (28) was 3.18, which is positively associated with the refractive degree of myopia.

The change in ACD corresponded inversely with lens thickness. After ciliary muscle paralysis, ACD deepened up to 0.18 mm due to the deformation and position shift of lens. Orinda’s study (31) found that LP decreased with age, which was consistent with our study. It is worth noting that researchers must pay attention to whether the measuring instruments used for the same parameter reported in different literature are consistent because the eye axis of ultrasonic measurement is shorter than that of optical low coherence reflectometry measurement, and the anterior chamber depth is also shallower (32).

Crystalline lens power plays an important role in the determination of refractive status (33). Most children are born hyperopic. With the eye growing, the losses of corneal and lens powers compensate for the axial elongation of the eye to keep refraction stable in a clustered distribution and gradually lead the refractive status toward emmetropia (34, 35). Differently, LP loss would last for the whole lifespan with its speed changed during different stages (26, 36), which is a critical component against myopic progression driven by the fast increase of AL. Both AL and LP determine refraction, and myopia develops if the rate of axial elongation exceeds the reduction in LP among adolescents. The crystalline lens power was calculated using Bennett’s formula and compared between different ages and refractive status. Our results of LP were higher than those mentioned above, and this may be largely related to differing inclusion criteria and instrumentation (37, 38).

Axial length and the AL/CCR ratio, respectively, explained 30.5 and 51.1% variance in SER. Similar to the previous research findings (20, 21) from school-aged children, the AL/CCR ratio seems to be having advantages over AL in contributing variance in SER, where AL accounted for 13 and 16% and AL/CCR for 31 and 45%, respectively. When both the AL/CCR ratio and LP were included, the model explained 59.2% of variance in SE, indicating that the crystalline lens also plays an important role in the process of emmetropization. Standardized longitudinal cohort study would have unique significance for the understanding of the changes of lens refraction during emmetropization and of the visual feedback mechanism related to regulation.

The MPOD for the entire group of studied subjects was 0.23 ± 0.08, and no statistically significant correlations were found between MPOD and SER, which is consistent with the results of previous studies (39, 40). We suspect that similar normal fundus among these groups accounts for no difference of MPOD. The MPOD in high myopia, however, deserves further investigation owing to the marked fundus changes. There is something that cannot be neglected. An accurate repeatable measurement of MPOD is vital in clinical diagnosis and treatment, as MPOD is influenced by various other factors, such as food habits (lutein supplementation), smoking, obesity, and macular diseases (40, 41). A follow-up study with a good design, larger sample size, and exclusion of other influencing factors is needed to determine the correlation between MPOD and SER.

There are several limitations to our study. First, we do not show enough data on eye parameters of emmetropic adolescents at different ages. Second, as this was a cross-sectional study, which means requiring a prospective longitudinal study to determine the anatomical changes of the eye as axial myopia progresses (especially regarding the relationship between LP and AL). However, our study relies on the best clinical eye centers and optometry centers in the country, and the accuracy of the data is guaranteed. Third, the internal mechanism between the LP and SER is not entirely clear. Further research with a wider range of SER should better interpret the changes of crystal refraction during emmetropization and explore the visual feedback mechanism related to regulation. Finally, our results might only be applicable to children aged 6–14 years.

The current results have the following clinical significance. First, what we know from the present literature concerns the ocular biometric characteristics of children recruited from general surveys or from sampling strategies. Second, we have studied the distribution of ocular biometric characteristics of mild hyperopia to mild myopia in children from the perspective of an optometry center having the best knowledge for the treatment of ametropic defects. In view of the reported stability of the epidemic of myopia, 1-year intervals would not likely cause significant cohort effects. In this case, cross-sectional data can be viewed and calculated as longitudinal data. Therefore, we can tentatively interpret that children recruited from this optometry center had a slower progression from hyperopia toward myopia. Third, scarce information has been reported about the relationship between the macular pigment optical density and spherical equivalent refraction, and no correlation was found between the macular pigment optical density and spherical equivalent refraction.

## Conclusion

In this work, we have studied the distributions of ocular biometric characteristics of mild hyperopia to mild myopia children from the perspective of optometry center rather than a sampling survey. We found that children from the optometry center had a slower progression toward myopia than those from previous sampling surveys, which was an informative finding for future myopia prevention. In addition, we have made correlation analysis between the macular pigment optical density and spherical equivalent refraction, though no correlation was found.

## Data availability statement

The raw data supporting the conclusions of this article will be made available by the authors, without undue reservation.

## Ethics statement

The studies involving human participants were reviewed and approved by Ethics Committee of the Eye Hospital of Wenzhou Medical University. Written informed consent to participate in this study was provided by the participants’ legal guardian/next of kin.

## Author contributions

CW contributed to the study conception and design. WQL and WSL performed material preparation, data collection, and analysis and wrote the first draft of the manuscript. All authors commented on previous versions of the manuscript and read and approved the final manuscript.
